# Association between type 2 diabetes and 5-year overall survival in early-stage pancreatic cancer: a retrospective cohort study

**DOI:** 10.7717/peerj.14538

**Published:** 2022-12-12

**Authors:** Zhiyin Tang, Wanfeng Xu, Mingming Zhang

**Affiliations:** 1 Department of Anesthesiology, Shengjing Hospital of China Medical University, Shenyang, China; 2 Department of Endocrinology, Shengjing Hospital of China Medical University, Shenyang, China; 3 Department of Pathology, Shengjing Hospital of China Medical University, Shenyang, China

**Keywords:** Diabetes mellitus, Pancreatic cancer, Glycemic control, Survival analysis, Asian continental ancestry group

## Abstract

**Background:**

This study examined the association between type 2 diabetes mellitus (T2DM) and 5-year overall survival (OS) in patients with pancreatic cancer (PC).

**Methods:**

This retrospective cohort study included patients diagnosed with stage I/II PC at Shengjing Hospital of China Medical University from January 2012 to December 2017. All patients had pancreatic ductal adenocarcinoma or its subtypes. The outcome was the 5-year OS rate based on data from the patient charts. Data analysis was performed using SPSS 22.0

**Results:**

A total of 238 patients were included: 72 with T2DM and 166 without T2DM. There were significant differences in blood glucose levels and OS between the two groups (all *P* < 0.05). The median OS was 11.4 (95% confidence interval CI [8.49–14.31]) months in the T2DM group and 16.3 (95% CI [12.44–20.16], *P* = 0.023) months in the non-T2DM group. After adjustment for confounders, T2DM was an independent factor affecting 5-year OS (*P* = 0.010). Compared with non-T2DM patients, T2DM patients had a higher risk of death (HR = 1.475, 95% CI [1.096–1.985]).

**Conclusions:**

T2DM is associated with 5-year OS in patients with PC.

## Introduction

Pancreatic cancer (PC) is a highly aggressive and intractable malignancy and one of the deadliest cancers, with poor prognosis and outcomes worldwide (Allemani et al., 2018; Rahib et al., 2014). Despite great advances in diagnostic and therapeutic strategies in recent decades, the 5-year overall survival (OS) in PC has not changed, while PC-attributable disability-adjusted life years (DALYs) more than doubled (GBD 2017 Pancreatic Cancer Collaborators, 2019). With a 5-year OS of 2–15%, PC might be the second deadliest malignancy in the next decade, especially in high-income countries (Arnold et al., 2019; Neoptolemos et al., 2018). Identification of novel biomarkers, laparoscopic surgical techniques, and neo-adjuvant chemoradiotherapy might offer opportunities to improve patient outcomes (Arnold et al., 2019). Still, non-specific early-stage symptoms and the retroperitoneal position of the pancreas limit routine assessments, leading to late-stage diagnosis and poor prognosis (McGuigan et al., 2018).

Previous studies indicated a 5-year survival of only 2% for patients with PC and that 80–85% of tumors are diagnosed at late and unresectable stages (Kamisawa et al., 2016). In addition, patients have evidence of local recurrence, metachronous metastasis, and high tumor chemoresistance reported in up to 80% of the cases (Neoptolemos et al., 2018; Poruk et al., 2013). Therefore, in addition to early detection, evaluating the risk factors is important for early treatment initiation. The nonmodifiable survival risk factors for PC include older age, a family history of cancer, African-American ethnicity, chronic pancreatitis, and diabetes, and the modifiable risk factors include smoking, obesity, and diet (Midha, Chawla & Garg, 2016). A previous study revealed that the gut microbiota affects survival in PC (Memba et al., 2017). In patients with familial risk factors, the odds of developing PC increase exponentially with the number of affected first-degree relatives (Becker et al., 2014), with the *Breast cancer gene 2* (*BRCA2*) and *Partner and localizer of BRCA2* (*PALB2*) mutations being the most common (Chen, Roberts & Klein, 2017).

Type 2 diabetes mellitus (T2DM) and hyperglycemia are well-established risk factors of PC, diagnosed in up to 85% of patients (Olakowska & Olakowski, 2021). PC manifestations can also mimic T2DM onset (Andersen et al., 2017). The risk of PC was shown to be two-fold higher in patients with T2DM, and HbA1c was proposed as a potential early biomarker of PC (McGuigan et al., 2018; Sharma et al., 2018). Patients with T2DM and PC also have a poor response to chemotherapy, larger tumors, and an elevated risk of death post-chemotherapy (Ma et al., 2019). Nevertheless, some studies failed to observe the significant effects of preoperative T2DM on the postoperative course (Deo et al., 2021) and prognosis (Balzano et al., 2016). A meta-analysis of 29 trials with 33 risk estimates suggested that T2DM is associated with reduced survival only in patients with resectable PC (Mao et al., 2015). Given the ambiguity of the available results and considering that most studies used a cutoff of 2 years for diabetes duration, studies with a longer follow-up are needed to examine the association between T2DM and the survival of PC patients.

Therefore, this study aimed to examine the association between T2DM and 5-year OS in patients with PC.

## Materials and Methods

### Study design and patients

This retrospective cohort study included patients diagnosed with PC at the Department of Pathology of Shengjing Hospital of China Medical University between January 2012 and December 2017. The inclusion criteria were (1) surgical resection and (2) diagnosis of stage I or II PC based on the American Joint Committee on Cancer eighth edition staging system (AJCC 8th edition). The exclusion criteria were (1) incomplete clinical history, (2) severe complications affecting OS, such as severe infection, hemorrhagic shock, intestinal fistula, respiratory or circulatory failure, or survival <1 month after surgery, or (3) unavailable follow-up. This study was approved by the Ethics Committee of the authors’ hospital, and informed consent was waived due to the retrospective nature of this study.

### Data collection and definitions

Patient demographics were obtained, *i.e*., age, sex, body mass index (BMI), smoking history, drinking history, hypertension history, diabetes history, tumor location, tumor differentiation degree, tumor stage, lymph node metastasis, surgical approach, blood glucose, carbohydrate antigen 199 (CA199) levels, and OS.

Hypertension was diagnosed according to the 2010 China Hypertension Prevention Guide as three measurements of blood pressure taken on different days with systolic pressure ≥140 mmHg and/or diastolic pressure ≥90 mmHg. Diabetes was diagnosed according to the 2009 diagnostic criteria of the American Diabetes Association (2009). The surgical methods included radical pancreaticoduodenectomy (HP), distal pancreatectomy or pancreatic body-tail resection, and other surgical methods (total pancreatectomy, extended pancreatectomy, and unknown surgery). OS time was defined from surgery to death using data from the patient charts. Patients with no specific date of death or date of loss to follow-up were excluded because OS could not be calculated.

### Outcomes

**Figure 1:**
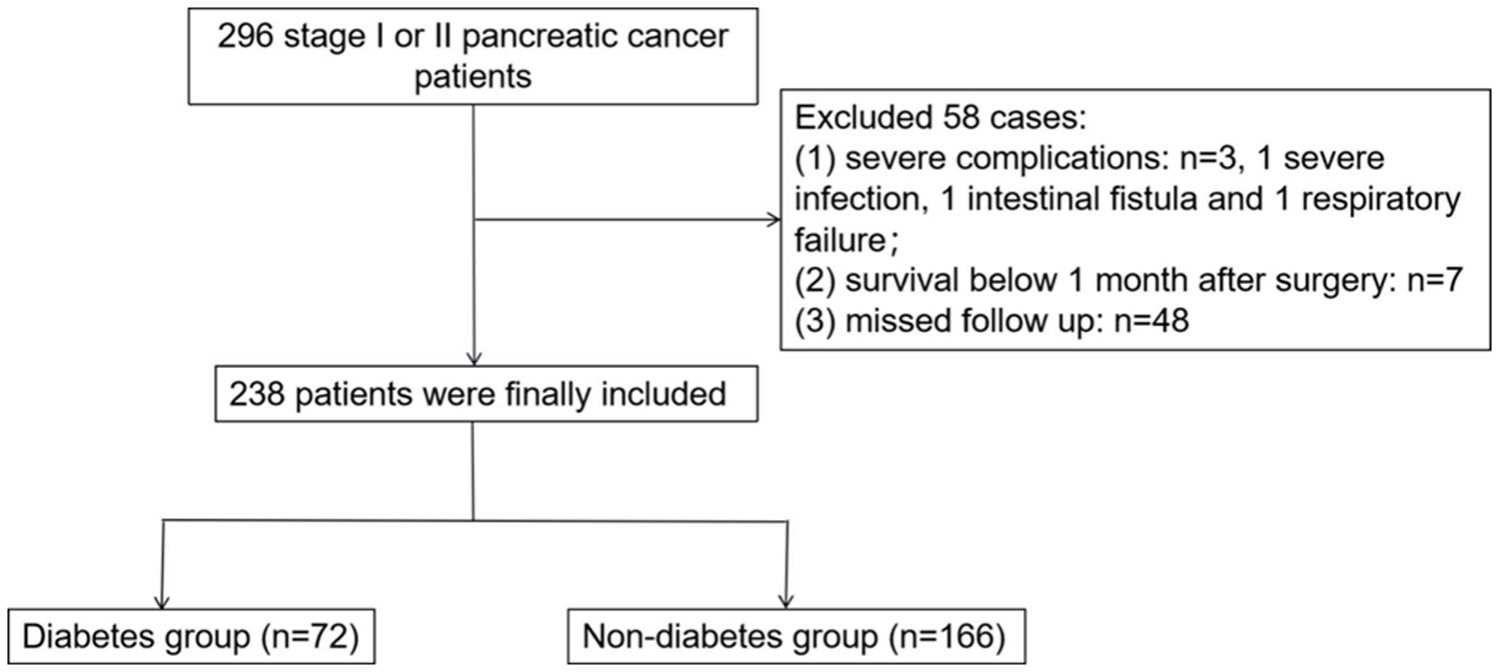
Study flowchart.

The outcome was the 5-year OS. The patients were grouped according to the presence of T2DM (Fig. 1).

### Statistical analysis

SPSS 22.0 (IBM, Armonk, NY, USA) was used for data analysis. The continuous variables were tested for normal distribution using the Kolmogorov-Smirnov test, and the continuous variables were found to be non-normally distributed. Therefore, they were presented as median (25^th^ and 75^th^ percentiles) and compared using the Kruskal-Wallis H-test. Categorical variables were expressed as *n* (%) and compared using the chi-square test or Fisher’s exact probability method. Survival was assessed using the Kaplan-Meier method, and the curves were compared using the log-rank test. A multivariable Cox regression analysis was used to examine the association between T2DM and 5-year OS after adjustment for covariates. The Forward: LR method was used to screen the variables. *P* < 0.05 indicated statistical significance.

## Results

There were 238 included patients: 72 with T2DM and 166 without T2DM (Fig. 1). There were significant differences in blood glucose, drinking history, and hypertension history between the two groups (all *P* < 0.05). In addition, the non-T2DM group had a longer OS than the T2DM group (*P* = 0.023). The detailed baseline information is shown in Table 1.

**Table 1:** Baseline data for pancreatic cancer cases with and without diabetes.

Clinical parameter	Diabetes cohort (*n* = 72)	Non-diabetes cohort (*n* = 166)	*p*
Age, years	62.00 (55.50, 66.75)	61.00 (53.00, 67.00)	0.485
BMI, kg/cm^2^	23.60 (21.55, 24.69)	23.09 (20.57, 24.85)	0.242
Sex			0.432
Male	43 (59.72%)	90 (54.22%)	
Female	29 (40.28%)	76 (45.78%)	
Smoking history			0.370
With	30 (41.67%)	59 (35.54%)	
Without	42 (58.33%)	107 (64.46%)	
Drinking history			0.001
With	24 (33.33%)	24 (14.46%)	
Without	48 (66.67%)	142 (85.54%)	
History of hypertension			0.005
With	30 (41.67%)	39 (23.49%)	
Without	42 (58.33%)	127 (76.51%)	
Tumor location			0.277
Head of pancreas	54 (75.00%)	134 (81.21%)	
Pancreatic body/tail	18 (25.00%)	31 (18.79%)	
Differentiation of tumors			0.319
High/Middle	54 (75.00%)	134 (80.72%)	
Low	18 (25.00%)	32 (19.28%)	
Lymph node metastasis			0.164
Yes	10 (14.71%)	35 (22.88%)	
No	58 (85.29%)	118 (77.12%)	
Tumor stage,			0.368
Phase I	44 (61.11%)	91 (54.82%)	
Phase II	28 (38.89%)	75 (45.18%)	
Surgical strategy			0.288
Radical pancreaticoduodenectomy	51 (70.83%)	129 (77.71%)	
Distal pancreatectomy	16 (22.22%)	32 (19.28%)	
Other surgical procedures*	5 (6.94%)	5 (3.01%)	
CA199			0.372
Normal	25 (34.72%)	48 (28.92%)	
High	47 (65.28%)	118 (71.08%)	
Blood glucose, mmol/L	8.93 (6.53, 11.36)	6.07 (5.44, 6.89)	<0.001
Overall survival, months	11.43 (6.29, 22.38)	16.35 (8.55, 34.61)	0.023

**Notes:**

* Other surgical procedures included total pancreatectomy, enlarged pancreatectomy and unknown surgery (surgical method not recorded for the case).

BMI, body mass index; CA199, Carbohydrate Antigen 199. Continuous data are presented as median (25^th^, 75^th^ percentiles). Categorical data are presented as *n* (%).

**Figure 2:**
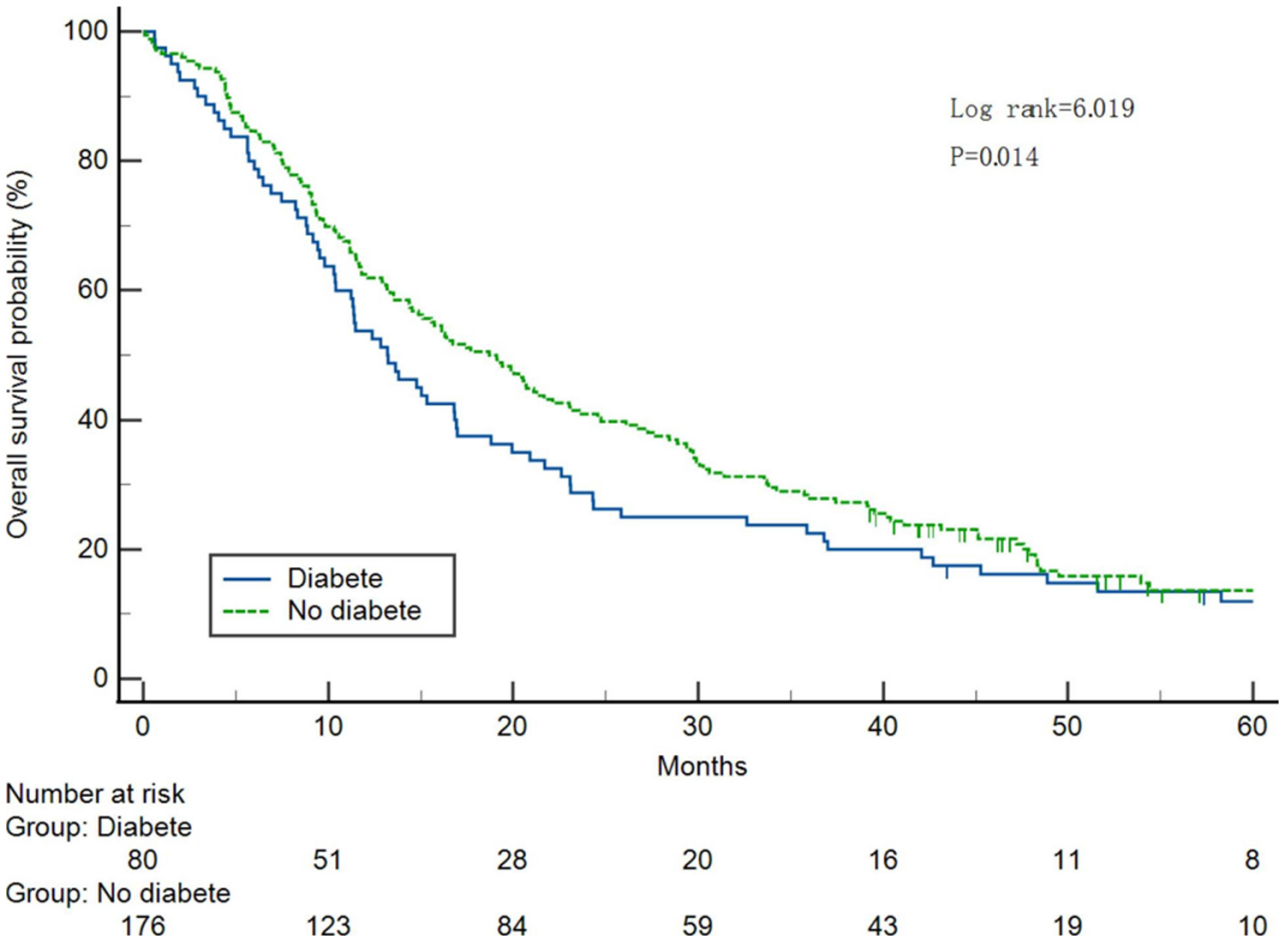
Analysis of 5-year survival in pancreatic cancer cases with or without diabetes.

Survival curves were plotted (Fig. 2). The median OS was significantly different between the two groups, with 11.4 months (95% CI [8.49–14.31]) and 16.3 months (95% CI [12.44–20.16]) in the T2DM and non-T2DM groups, respectively (*P* = 0.023).

The crude (unadjusted) HR of T2DM for 5-year OS was 1.52 (95% CI [1.13–2.05], *P* = 0.005). After adjustment for covariates (sex and tumor differentiation), T2DM was an independent factor affecting 5-year OS (*P* = 0.010). Compared with non-T2DM patients, T2DM patients had a higher risk of death (HR = 1.475, 95% CI [1.096–1.985]) (Table 2).

**Table 2:** Multivariable analysis of overall survival.

	Univariable		Multivariable	
	HR (95% CI)	*P*	HR (95% CI)	*P*
Age	1.001 (0.986–1.016)	0.925		
BMI	0.990 (0.946–1.035)	0.650		
Sex				
Male	Reference			
Female	0.698 (0.530–0.919)	0.010		
Smoking history	1.075 (0.815–1.416)	0.610		
Drinking history	1.124 (0.806–1.567)	0.492		
History of hypertension	1.000 (0.744–1.344)	0.999		
Tumor location				
Head of pancreas	Reference			
Pancreatic body/tail	0.998 (0.711–1.399)	0.989		
Tumor differentiation				
High/moderate	Reference			
Low	1.645 (1.187–2.279)	0.003	1.589 (1.146–2.204)	0.006
Lymph node metastasis	0.722 (0.514–1.015)	0.061		
Tumor stage				
Phase I	Reference			
Phase II	1.119 (0.854–1.465)	0.415		
Surgical approach		0.996		
Radical pancreaticoduodenectomy	Reference			
Distal pancreatectomy	0.994 (0.707–1.398)	0.973		
Other surgical procedures	0.972 (0.496–1.905)	0.934		
CA199				
Normal	Reference			
High	1.170 (0.875–1.566)	0.290		
Type 2 diabetes	1.524 (1.134–2.048)	0.005	1.475 (1.096–1.985)	0.010

## Discussion

The present study suggested that 5-year OS was longer in patients with PC without T2DM, while T2DM was associated with 5-year OS in patients with early-stage PC (AJCC 8^th^ edition stage I and II). Careful management of glucose levels in patients with PC might increase OS.

Whether T2DM impacts the survival of patients with PC remains controversial, with adequately powered randomized trials reporting diverging results. This study found that T2DM significantly influenced the survival of patients with PC. Among the factors that might affect the demonstrated relationship and should be considered to compare the current and previously reported findings, obesity, race, glucose control, and differences in methods used for T2DM screening are the most discussed. Previous studies proposed a sound theory according to which the association between T2DM and survival to PC is indirect and mediated *via* obesity, with metabolic syndrome playing a role in both diseases (Genkinger et al., 2015). It was shown that the metabolic syndrome-related gene guanosine monophosphate synthetase (GMPS) could predict prognosis in pancreatic ductal adenocarcinoma (PDAC) (Cai et al., 2021). On the other hand, in a survival analysis by Téoule et al. (2020) in 76 fully-matched pairs obtained after the screening of 553 patients, obesity had no effects on OS and postsurgical complications in patients with PC. Furthermore, a high BMI among patients with PC was associated with improved OS, particularly in individuals who were administered chemotherapy (Fu et al., 2021). This study included patients with BMI values from 21.6 to 24.9 kg/m^2^, and T2DM was associated with survival to PC independently of BMI, which at least partly contradicts the obesity theory and confirms that other factors besides the metabolic syndrome contribute to the lower survival rates observed in patients with T2DM and PC.

Another factor influencing the results is demographics, particularly the percentage of nonwhite or Asian patients. For example, a study by Kellam & Yim (2018) performed in Wales showed that the median OS was slightly lower in DM patients compared with non-diabetic patients (12.0 months, 95 CI [5.9–18.1] months *vs*. 13.0 months, 95% CI [8.6–17.3] months). Nevertheless, the sample size (131 patients with PC, including 58 with T2DM) of the latter study was not statistically significant (*P* = 0.334). Another recent analysis included patients from the RTOG 9704 trial (Bitterman et al., 2021) and showed that T2DM, insulin use, and obesity had no associations with OS in patients with PC but noted a significantly lower survival rate in nonwhite patients. Finally, in a study by Iizumi et al. (2019) that included exclusively Asian participants, progression-free survival (PFS) and OS were markedly shorter in patients with T2DM and metastatic PC. Here, all participants were of Chinese Han nationality, and their features of T2DM might differ from those of Western patients, including an elevated risk of diabetes at a lower BMI and a more evident association with malignancy, as previously described (Noto, Tsujimoto & Noda, 2012). Indeed, the Chinese generally have a lower BMI than westerners (Oakkar et al., 2015), but the prevalence of T2DM in China is above the worldwide values (Collaboration NCDRF, 2016; Wang et al., 2021), indicating some discrepancies or differences in the pathogenesis of metabolic syndrome and T2DM that might influence the relationship between T2DM and PC between Asians and Europeans and Americans (Ma & Chan, 2013). A study suggested that Asians might have a lower ability to secrete insulin, and insulin resistance is possibly not the driver of T2DM in Asians (Narayan & Kanaya, 2020). The influence of these differences on PC will have to be examined more closely within the same study. Beyond differences in genetics and metabolism, lifestyle habit differences should also be examined. Indeed, red meat, processed meat, and refined sugar consumption are higher in Europe and America, and these factors could influence both T2DM (Hu, 2011) and PC (Kamisawa et al., 2016; Kleeff et al., 2016; McGuigan et al., 2018), especially the glycemic index (Turati et al., 2015).

Compared with hypoglycemia, hyperglycemia was shown to promote the *in vitro* proliferation, migration, and invasion of PC cells (Yu, Zhang & Zhang, 2021). Therefore, there is a possibility that glycemic control might be a different factor contributing to the survival to PC in each patient (Alpertunga et al., 2021; Karlin et al., 2018). This theory was partly confirmed by a multivariable analysis in which recent T2DM onset had an independent association with PDAC relapse (HR = 1.45, 95% CI [1.06–1.99]) (Balzano et al., 2016). In addition, the negative impact of DM on 3- and 5-year disease-free survival was demonstrated with no marked effect on the postsurgical course in patients with pancreatic and periampullary adenocarcinoma (Deo et al., 2021), implying that the processes determining the impact of T2DM might be delayed. Long-standing T2DM is associated with a shorter PFS, while in the early T2DM group, both PFS and OS differ from patient to patient (Bitterman et al., 2021). In this study, there was a baseline difference in mean blood glucose levels, with 8.93 (6.53, 11.36) mmol/L in the T2DM group *vs*. 6.07 (5.44, 6.89) mmol/L in the non-T2DM group (*P* < 0.001), suggesting that a better glycemic control might increase the 5-year survival rate in patients with PC, which deserves further investigation.

This study had some limitations. First, due to its retrospective nature, some selection bias might be present. Second, this single-center study included patients treated at the same study site who may have specific features related to the same region, particularly regarding glucose control and T2DM management. Third, because many patients did not undergo T2DM screening before PC diagnosis, HbA1c was missing in too many patients to be analyzable. Finally, only patients with BMI under 26 kg/m^2^ were included in the present study, which limits the ability to discuss the effects of obesity and metabolic syndrome on PC. Further high-level evidence is needed from large-sample, multicenter, prospective, randomized clinical trials.

## Conclusion

T2DM is associated with 5-year OS in patients with early-stage PC, significantly reducing the 5-year OS and OS rates. These results contribute to the ongoing discussion regarding the impact of diabetes on the survival to PC, partly contradicting the obesity theory and drawing more attention to the role of hyperglycemia in the pathogenesis of PC relapse. Asian/nonwhite race, especially glycemic control, and T2DM duration might affect the demonstrated relationship and should be further explored in future studies.

## Supplemental Information

10.7717/peerj.14538/supp-1 Supplemental Information 1 Raw data.

## Additional Information and Declarations

### Data Availability

The following information was supplied regarding data availability: The raw measurements are available as a Supplemental File.
